# Influences of post-implementation factors on the sustainability, sustainment, and intra-organizational spread of complex interventions

**DOI:** 10.1186/s12913-022-08026-x

**Published:** 2022-05-17

**Authors:** Yuting Song, Lauren MacEachern, Malcolm B. Doupe, Liane Ginsburg, Stephanie A. Chamberlain, Lisa Cranley, Adam Easterbrook, Matthias Hoben, Jennifer Knopp-Sihota, R. Colin Reid, Adrian Wagg, Carole A. Estabrooks, Janice M. Keefe, Tim Rappon, Whitney B. Berta

**Affiliations:** 1 School of Nursing, Qingdao University, 308 Ningxia Road, Qingdao, 266071 Shandong Province China; 2 Faculty of Nursing, University of Alberta, Edmonton Clinic Health Academy, 5-305, 11405 87 Ave, AB T6G 1C9 Edmonton, Canada; 3 Management & Evaluation, Institute for Health Policy, University of Toronto, 155 College Street, Suite 425, Toronto, ON M5T 1P8 Canada; 4 Max Rady College of Medicine, University of Manitoba, 408-727 McDermot Avenue, Winnipeg, MB R3E 3P5 Canada; 5 School of Health Policy & Management, York University, Stong College 353, 4700 Keele Street, Toronto, ON M3J 1P3 Canada; 6 Department of Family Medicine, University of Alberta, 6-50 University Terrace, Edmonton, AB T6G 2T4 Canada; 7 Lawrence S. Bloomberg Faculty of Nursing, University of Toronto, 155 College Street Suite 130, Toronto, ON M5T 1P8 Canada; 8 Centre for Health Evaluation and Outcome Sciences, St. Paul’s Hospital, 588 – 1081 Burrard Street, Vancouver, B.C. V6Z 1Y6 Canada; 9 Faculty of Health Disciplines, Athabasca University, 1 University Drive, Athabasca, AB T9S 3A3 Canada; 10 School of Health and Exercise Sciences, Faculty of Health and Social Development, University of British Columbia – Okanagan campus, 1147 Research Road, Kelowna, BC V1V 1V7 Canada; 11 Department of Medicine, University of Alberta, 1-198 Clinical Sciences building, 11350 – 83Avenue, Edmonton, AB T6G 2P4 Canada; 12 Nova Scotia Centre On Aging, Mount Saint Vincent University, McCain 201F, 166 Bedford Hwy, Halifax, NS B3M 2J6 Canada

**Keywords:** Implementation Science, Caregiving – Formal, Management, Long-term Care

## Abstract

**Background:**

Complex interventions are increasingly applied to healthcare problems. Understanding of post-implementation sustainment, sustainability, and spread of interventions is limited. We examine these phenomena for a complex quality improvement initiative led by care aides in 7 care homes (long-term care homes) in Manitoba, Canada. We report on factors influencing these phenomena two years after implementation.

**Methods:**

Data were collected in 2019 via small group interviews with unit- and care home-level managers (*n* = 11) from 6 of the 7 homes using the intervention. Interview participants discussed post-implementation factors that influenced continuing or abandoning core intervention elements (processes, behaviors) and key intervention benefits (outcomes, impact). Interviews were audio-recorded, transcribed verbatim, and analyzed with thematic analysis.

**Results:**

Sustainment of core elements and sustainability of key benefits were observed in 5 of the 6 participating care homes. Intra-unit intervention spread occurred in 3 of 6 homes. Factors influencing sustainment, sustainability, and spread related to intervention teams, unit and care home, and the long-term care system.

**Conclusions:**

Our findings contribute understanding on the importance of micro-, meso-, and macro-level factors to sustainability of key benefits and sustainment of some core processes. Inter-unit spread relates exclusively to meso-level factors of observability and practice change institutionalization. Interventions should be developed with post-implementation sustainability in mind and measures taken to protect against influences such as workforce instability and competing internal and external demands. Design should anticipate need to adapt interventions to strengthen post-implementation traction.

**Supplementary Information:**

The online version contains supplementary material available at 10.1186/s12913-022-08026-x.

## Background

Healthcare systems are increasingly conceptualized as complex adaptive systems that warrant complex solutions to improve care processes and practices [1]. Solutions are themselves frequently complex evidence-based interventions to introduce and support practice changes, requiring considerable effort and resources to implement. Researchers underscore the importance and challenges of sustaining practices, benefits, and outcomes introduced through complex interventions [2]. Most changes introduced through interventions subside over time [2, 3] – even highly implementable changes like prescribing practices after cardiac events [4].

Intervention sustainability is among “*the most significant translational research problems of our time*” [5], but sustainability-associated phenomena are persistently under-researched [2]. Implications of failing to sustain processes and outcomes of practice change initiatives are numerous. Failure (1) wastes financial and human resources [6]; (2) makes improvements short-lived, detracting from care quality and increasing variation [7]; (3) leads to inequities in health services access and quality [6, 7]; (4) diminishes probability of spreading beneficial solutions; and (5) reduces morale and belief in improvement initiatives [8].

### Study motivation

In understanding how to *implement to sustain*, most work addresses influences of peri-implementation factors (salient during the implementation phase) on sustainability. These factors include supports from implementers and features of pre-implementation context [2, 9–11]. Our premise is that *influences of* post*-implementation factors may be just as, or more, important to longer-term sustainability – particularly with well-established challenges to sustaining behavior change in notoriously intractable social settings such as healthcare* [12]*.* We argue that post-implementation factors are likely to prevail and gain salience after implementation supports are removed. While some post-implementation factors may not directly affect continued application of new practices, others may have profound influence. Factors may support new processes and practices, erode changes during implementation, or reinforce reversion to old ways of doing [12]. Withdrawal or redirection of management support, or loss of staff with knowledge of a practice change, may lead to knowledge loss and organizational forgetting.

Our study was further motivated by two other major literature gaps. First, the related phenomena of sustainability, sustainment, and spread (defined below) have been studied separately, or sustainment and sustainability are conflated. Little is known about whether factors that influence one phenomenon influence others. Second, how sustainability, sustainment, and spread are related is understudied and important to advancing sustainability science [5, 13].

### Defining sustainment, sustainability, and spread

The terms sustainment, sustainability, and spread lack universal definitions [13]. In the implementation science literature, sustainment and sustainability are considered post-implementation phenomena of concern once (peri)implementation supports are withdrawn or exhausted. Common supports are research funding for coaching and facilitation, access to clinical and other expertise, training, and financial incentives. Chambers et al. (2013) [14] usefully distinguish sustainment and sustainability as related but distinct aspects of continued knowledge use. *Sustainment* occurs when stakeholders continue to enact core processes and related behaviors originally conveyed through an intervention. *Sustainability* is continuation of key benefits or outcomes from the intervention. We refer to *spread* as uptake or diffusion of an intervention (or core components) from its original context to intra- or inter-organizational contexts [15]. While spread is often considered conceptually separate from sustainment and sustainability, we contend that it is a logically linked related phenomenon. If change processes are not sustained, or benefits cease to be realized, a change initiative is unlikely to spread [16]. We refer to sustainment, sustainability, and spread collectively as *sustainability phenomena*.

### What we know about sustainability phenomena and complex interventions in healthcare

Stirman and colleagues’ (2012) [2] critical review of the literature on sustainability of evidence-based programs and practices in healthcare settings highlighted four categories of factors that influence continuation of procedures and outcomes: (1) organizational contexts for practice change interventions (e.g., culture, leadership, structure); (2) internal and external capacity (e.g., resources, support, champions); (3) processes (e.g., training, engagement); and (4) aspects of new programs or practices themselves (e.g., fit, modifiability). These factors are influential peri-implementation and influence sustainability phenomena post-implementation. Stirman et al. (2012) [2] noted lack of consideration of sustainability dynamics. Most studies they reviewed considered sustainability at one time point post-implementation, for static presence/absence/extent of peri-implementation factors. Stirman et al. (2012) [2] concluded that sustainability research needs careful critical development, including enhanced conceptual clarity on sustainability and concerted examination of dynamics of contexts where sustainability is a concern.

More recent findings align with the categories of Stirman et al. (2012) [2]. Important peri-implementation factors that influence sustainability are intervention design and delivery, intervention process, people involved, resources, organizational setting, and external environment [13]. Proctor and colleagues (2015) [17] highlighted the need for definitional and conceptual consistency within the sustainability literature. They sought perspectives of sustainability researchers and stakeholders to identify important issues for sustainability research. They highlighted two priority areas: testing how intervention properties and adaptations affect sustainability, and identifying contextual factors that influence sustainability. They noted the importance of distinguishing between influences of factors on implementation and post-implementation.

Lennox and colleagues (2020; 2018) [8, 13] reviewed approaches to planning, assessing, or evaluating continued intervention activities and benefits. Their consolidated summary of sustainability determinants in these approaches is highly consistent with Stirman et al. (2012) [2]. They usefully identified recent empirical exploration of sustainability dynamism, noting a detectable “*shift in [researchers’] perspectives from sustainability as an outcome to sustainability as an ongoing process*” ([13], p. 12). About one third of identified approaches draw on theories that view sustainability as an ongoing process [8, 13]. Those relying on complexity theory emphasize interactions among intervention, organizational context, and broader context. Those relying on ecological theory propose ongoing intervention adaptation as important to maintaining fit with constantly changing inner and outer contexts. Those referring to open systems theory posit that broader environment influences organizational context of the intervention, requiring constant organizational adaptations to sustain the intervention. In all theoretical perspectives, the dynamic view of sustainability calls for continuous intervention improvement and adaptation and (where feasible) its inner and outer contexts [18]. These perspectives depart markedly from traditional linear views that treat sustainability as a static outcome.

While emerging dynamic perspectives on sustainability are promising and much more likely to reflect the complex reality of sustaining practice change over time, none of this work adequately considers changing intervention contexts. Assuming that factors active during implementation are equally active and effective post-implementation is counter-intuitive (and possibly naïve). The literature is silent on specific influences of post-implementation factors on sustainability.

### Sustainability phenomena of the SCOPE (Safer Care for Older People in Residential Environments) intervention

Our study is part of the Sustainment, Sustainability, and Spread Study (SSaSSy) [16] of a complex quality improvement (QI) initiative, SCOPE (ClinicalTrials.gov NCT03426072) [19]. Supplementary Fig. 1 shows the different stages of SCOPE, data collection for the current study, and SSaSSy. Our focus is sustainability phenomena in CHs in the province of Manitoba, Canada, where SCOPE was implemented during 2016–2017. We report more details of the SCOPE implementation in Manitoba elsewhere [20].

In Canadian CHs, care aides (unregulated direct care workers) account for the largest workforce and provide up to 90% of direct care for older adults [21]. SCOPE is a complex intervention to empower care aides to lead a QI initiative on their CH unit, with facilitated support. SCOPE has four components (Supplementary Fig. 2) [20, 22]. (1) Learning Congresses led by experienced quality advisors convey key *QI skills and processes* identified in the Institute for Health Improvement’s collaborative model (Institute for Healthcare Improvement, 2003) [23]. Interim action periods of Plan-Do-Study-Act (PDSA) cycles provide experiential QI learning. Small PDSA change cycles, enacted by collaborative teams and emphasizing pre-post measurement, gauge effects of small-scale practice change/QI initiatives. (2) SCOPE introduces a *team QI approach*. Care aides lead teams of 3–4 CH staff. (3) SCOPE formalizes *internal team support*, with Team Sponsors (unit managers) and Senior Sponsors (CH managers) mentoring and supporting QI activities of care aides. (4) Quality advisors provide *external team supports*, with ongoing coaching and guidance to Sponsors and QI teams over PDSA cycles between Learning Congresses.

SCOPE’s key components convey its core elements (processes, behaviors) designed to deliver key benefits (Table 1). Core elements are essential to sustain, as “*most closely associated with desired health benefit*s” ([2], p. 10). Each care aide-led QI team selected one of three clinical areas for their project focus: responsive behaviors, pain, or mobility. SCOPE’s intended key benefits (Table 1) are care aides’ QI capacity and leadership, use of best evidence in their practices, improved quality of work life, and enhanced work engagement. Research in work psychology [12] suggests that SCOPE’s influence on outcomes for work autonomy, empowerment, and engagement is likely to influence downstream work performance, including improved quality of resident care.

**Table 1:** SCOPE Core Processes and Key Benefits

**Core Elements, Processes, and Behaviors**
(Learn) QI skills and processes
(Use) Care aide-led team-based approaches
(Develop) Internal support from Sponsors: mentoring and supporting
(Provide) External support from quality advisors: coaching and guiding
**Key Benefits and Outcomes**
(Enhanced) QI capacity
(Enhanced) QI leadership
Use of best evidence in practice
(Improved) Care aide quality of work life
(Enhanced) Care aide work engagement

A SCOPE pilot was conducted in Alberta and British Columbia CHs (2010–2011) [24]. We examined sustainability and spread of SCOPE-related QI activities in 7 participating homes at 6 and 12 months post-implementation [25]. We did not differentiate peri- and post-implementation factors or sustainment and sustainability, but we identified factors influencing continued processes (sustainment) and sustained benefits (sustainability) up to 12 months post-implementation: turnover of key team members (care aides), facilitative team dynamics, observable improvements from SCOPE, strong management support of QI teams, buy-in from other CH staff, and (in)adequate resources [25]. Factors contributed to absence of continued processes (sustainment), sustained benefits (sustainability), or spread: lack of resources (financial, human) in one CH, leadership change in another, and competing government priorities in the third. These findings on short-term sustainability suggested factors warranting further exploration in this study.

### Study aims

Our overarching goal is to understand post-implementation factors that influence dynamic longer-term sustainment, sustainability, and spread of complex care interventions. In this exploratory study, we aimed to explore post-implementation factors that influenced the sustainment, sustainability, and spread of the SCOPE intervention in the complex environments of Canadian care homes (CHs; long-term care homes). Our research question was: What were unit and care home managers’ perspectives of multi-level *post*-implementation factors that influenced the continuation of core SCOPE elements and key benefits/impacts? The SCOPE intervention has been evolving as this research team developed [22], piloted [24], and implemented [20] the intervention. Therefore, our research team has the unique opportunity to contribute to knowledge about the dynamism of sustainability phenomena.

## Methods

We used secondary data that were originally collected in the preparation stage of SSaSSy to assess the post-implementation continuation of SCOPE-related QI activities in care homes and to inform the refinement of SSaSSy (Supplementary Fig. 1). We focused on post-implementation factors that unit and CH managers (Team and Senior Sponsors) identified as instrumental to continued or discontinued use of SCOPE’s core elements and benefits (Table 1).

### Study design

We employed a qualitative, descriptive research design [26] to explore factors influencing SCOPE sustainability phenomena two years post-implementation. This design allowed us to stay close to the data with low levels of researcher interpretation or inference [26]. We collected data through small group interviews by telephone (May to July 2019), two years after SCOPE’s implementation in Manitoba (2016–2017).

### Setting and participants

SCOPE was implemented in 7 CHs in Winnipeg (Manitoba’s largest urban center). While multiple groups of staff (care aides, regulated staff, managers) participated in the SCOPE implementation, in this study we focused on managers’ perspectives of sustainability phenomena post-implementation. We invited all unit and CH managers to participate via email; 11 managers from 6 of 7 CHs agreed. We undertook 6 small group interviews (1 to 3 managers per interview), one for each CH participating.

### Guide for small group interviews

We based our semi-structured interview guide (Supplementary File 1) on literature on QI program sustainability in healthcare settings [2, 8, 13, 17] and our prior work [25]. We elicited participants’ descriptions of their experiences during SCOPE, continued QI activities post-implementation, post-implementation factors influencing sustainment (dis/continuation of behaviors) and sustainability (dis/continuation of outcomes) in the QI unit, and inter-unit spread.

### Data collection and preparation

LM sought written informed consent from potential participants prior to each interview. LM and WB conducted telephone interviews of up to 45 min, each with manager participants from the same care home. Interviews were audio-recorded, professionally transcribed, and de-identified before analysis.

### Data analysis

We used inductive thematic analysis to identify data patterns and themes [27, 28] and Microsoft Word to support the analysis. Table 2 details the analysis steps, including familiarizing with the data, generating, and applying coding scheme, generating themes, and refining themes. When developing and refining the coding scheme, we organized observed factors into three levels: (1) micro-level factors relating to individuals, teams, and their interactions; (2) meso-level factors relating to unit and CH; and (3) macro-level factors relating to CH operating environment or systems.

**Table 2:** Steps of thematic analysis with authors’ roles

Steps	Description
Familiarize with the data	The analysis team (YS, LM, WB) read through the full transcripts of the six interviews
Generate and apply coding scheme	The team undertook an iterative process between codes and raw data to develop a coding scheme (1) LM randomly selected two of the six transcripts. Drawing on factors influencing sustainability phenomena from sustainability literature, the team independently coded the two transcripts, using open coding by assigning codes to chunks of data (2) The team compared coding via regular group discussions, clarified ambiguities and inconsistencies until they reached consensus of a preliminary coding scheme (3) Using the coding scheme while being open to emerging codes, the team independently coded two additional transcripts (4) The team went through (2) again, finished coding all six transcripts, and refined the coding scheme (5) The team discussed the coding scheme with authors LG, MD, AW and LC and agreed on a final set of coding scheme (6) The team independently reviewed the coding of all transcripts for consistency with the final coding scheme
Generate themes	Based on the coding, YS and LM prepared extensive memos for each code and attended to the relationships between individual codes. Patterns were identified and themes emerged in the process. The team conducted rounds of discussions and revisions of the themes
Refine themes	Authors LG, MD, AW and LC provided input to the refinement of the themes identified through the data analysis

For qualitative rigor [29], multiple researchers worked closely in analysis and recorded major decisions and findings in extensive memos. A three-member team (YS, LM, WB) led the data analysis; this team included a health services research PhD student, a nursing postdoctoral fellow, and a health services researcher. Several other authors (LG, MD, AW, LC) contributed to the identification and differentiation of themes and to the refinement of them.

## Results

### General observations on post-implementation sustainment, sustainability, and spread

#### Sustainment of core elements

Two years after SCOPE, Sponsors (managers) from 5 of 6 teams (CH1, CH3-6) reported continuation of several SCOPE-related QI activities (Supplementary Table 1) for core processes and behaviors. Only CH2 reported complete cessation. Sustained processes and behaviors included: QI processes like PDSA cycles to identify and trial change ideas (CH3); team-based approaches to QI (e.g., regular staff huddles [CH1]); QI ideas identified during SCOPE implementation (e.g., individualized mobility reminder cards [CH1], music to reduce responsive behaviors [CH4,5]); and QI tools to continually assess progress (e.g., safety crosses, aggressive behavior assessment tool [CH4]). Some units had well-preserved SCOPE team membership and core elements (Table 3 Quote 1).

**Table 3:** Summary of quotes for sustainability phenomena and themes

Sustainability phenomena and themes	Quote #	Quote
Sustainment of core elements	1	*“There are still three of [5 of] those team members who are working on SCOPE. They're still using some of the…measurement tools that they developed. They're still focusing on disruptive behaviors.”* [Team Sponsor, CH4]
Sustainability of key benefits	2	*“She is looking for opportunities to improve. She's giving suggestions, what approach will work and does not work for this particular special-needs unit, and the team listens to her suggestions, and we trial it, and if it will work, then we put it into place.”* [Senior Sponsor, CH3]
	3	*“We just have gone through … an inter-RAI [Resident Assessment Instrument] quality of life residents survey, their satisfaction survey for this year… and actually some of what we see in there, we actually believe that the work that the SCOPE team did back in 2016–2017 might be utilized to address some of the areas where we see room for improvement based on those results… my vision is to bring healthcare aides back into that process of revisioning how we implement that on the floor… you would hope that you implement your new initiative using the tools that our healthcare aides developed.”* [Senior Sponsor, CH6]
Intra-organizational spread	4	*“It almost has become second nature for people… The walk that we've been doing and the cards [indicating residents’ mobility level] that they [care aides] rolled out are on all the floors.”* [Senior Sponsor, CH1]
Theme 1: Adaptive capacity of care aides and adaptability of the intervention facilitate sustainment	5	*“If they [care aides] have hurdles, at the end of shift, they sit and say like, ‘This one [resident] we have this problem and we tried a few things, but it still doesn't work.’ Then they're trying different people, not from one group only… And then they're getting a better understanding of what can be changed. It's an ongoing process.”* [Senior Sponsor, CH3]
Theme 2: Facilitative formal leadership of Sponsors is important to sustainment and sustainability	6	*“It's not like, ‘I don't think [it] is a good idea … it will be too expensive. Bye, see you later.’ Even if I [am] afraid that this is not going to work, still, I'll give her the opportunity to try… it's not coming from me prescribing what to do, it's actually coming from her, and she has some control [over] it.”* [Senior Sponsor, CH3]
	7	*“The good thing …is, it makes the healthcare aides be the leader, and I'll be the follower…I think those are the strengths that I think I can offer to them. It's just like, ‘You guys, you started this. You can see some improvements. We can do this…’”* [Team Sponsor, CH4]
Theme 3: Social dynamics among Sponsors and care aides are important to sustainment	8	*“If somebody [care aides] needs something, they will come to her [a particular care aide] and say like, ‘You know how to talk to these people in the office.’ … because she developed some communication with us, the trust …”* [Senior Sponsor, CH3]
	9	*“We do tours of newcomers [older adults and/or family members] coming in and when they see the mobility sign, they make comments on it. We need to pass those compliments on to the SCOPE team themselves.”* [Senior Sponsor, CH1]
Theme 4: Culture of innovation and change is associated with sustainment	10	*“I had been in the recreation department beforehand, and there [are] always days where everyone's trying to think, ‘What can we do differently? How can we improve the process and basically helping the residents?’ We're very resident focused here, so it's just the natural [way].”* [Team Sponsor, CH1]
Theme 5: Structural mechanisms are associated with sustainment and sustainability	11	*“I think talking about it, about their work at all of our different meetings and making sure that they receive the emails talking about how great their work is. These sort of piquing other people's curiosity to know what it's all about and making that seem to feel special, like appreciating their work.”* [Senior Sponsor, CH1]
Theme 6: Institutionalized practice changes contribute to sustainment and spread	12	*“The walk that we've been doing and the cards that they rolled out [during SCOPE] are [still used] on all the floors and it's just a little prompt as you enter their room, [about] what kind of mobility is required and … a reminder for the staff to do that.”* [Senior Sponsor, CH1]
Theme 7: Workforce stability influences sustainment		No quotes cited in the text
Theme 8: Competing internal demands detract from sustainment	13	*“In everyday work, there's always something that comes in that you’ve got to do. As a leader, that [SCOPE project] was one of the things that just fell off.”* [Senior Sponsor, CH2]
	14	*“You have to pick a few things and say this is what we're going to work on … If we could say, have the healthcare aide teams working on initiative X because that's part of our larger quality operational plan for the year … I think there could be a good synergy there … We're not there yet … we're still entrenched in all places doing things”.* [Senior Sponsor, CH6]
Theme 9: Observable benefits are associated with sustainment, sustainability, and spread	15	*“In order for us to roll it out to others, we had had to show that, yes, if we continue to keep these folks mobile, it's going to make all these other benefits with our residents, and that's when it makes our life here as caregivers [better]. That was the buy-in right there.”* [Team Sponsor, CH1]
	16	*“If they don't see an improvement, they don't want to [be] involved [in] it. … if they see some changes in staff they get more interested, they ask more questions. That's how we do spreading the change which is we do some posters, storyboards, and memos.”* [Team Sponsor, CH4]
Theme 10: Alignment with system imperatives affects sustainment and sustainability	17	*“… The fact that … we've been closing out the emergency [departments in hospitals, in their jurisdiction] and everything, and cutting off funding, so we don't have enough staff sometimes. For that reason, you can't really give quality care if you don't have enough resources.”* [Team Sponsor, CH4]
	18	*“Yes, this is important. We got it, we need to work on this. But my operational priorities, I also have to look at what the long-term care program and [the overseer] is identifying as their priorities for the program. Because I, as an organization, need to support the larger system and how I fit with that.”* [Senior Sponsor, CH6]

#### Sustainability of key benefits

Sponsors reported sustained SCOPE benefits over the two years post-implementation (Supplementary Table 1). Improved quality of work life across sites CH1 and CH3–6 manifested as enduring care aide empowerment and increased self-efficacy. Sponsors remarked on care aides’ empowerment, demonstrated through behaviors outside traditional roles (e.g., initiating QI discussions, continuing to suggest specific change ideas). The CH3 Senior Sponsor described one care aide continuing to lead QI initiatives post-implementation (Table 3 Quote 2). Several Sponsors described shifts in their perceptions of care aides’ QI capacity, which persisted post SCOPE. Sponsors involved care aides in more QI initiatives, likely influencing sustainability of key benefits including work engagement. The CH6 Senior Sponsor saw value of care aides’ SCOPE work and planned to involve them in another QI project (Table 3 Quote 3).

#### Intra-organizational spread

Sponsors from three CHs (CH1,4,5) reported that SCOPE-related activities spread to additional units (Supplementary Table 1). After a SCOPE initiative to reduce responsive behaviors by reducing over-stimulation during meals, the CH5 Team Sponsor noted: *“The other three units on the other side of the building are now being more aware of content on the television, music during mealtimes.”* A SCOPE initiative to improve resident mobility spread to all of CH1 (Table 3 Quote 4).

### Post-implementation factors influencing sustainability phenomena

**Fig. 1:**
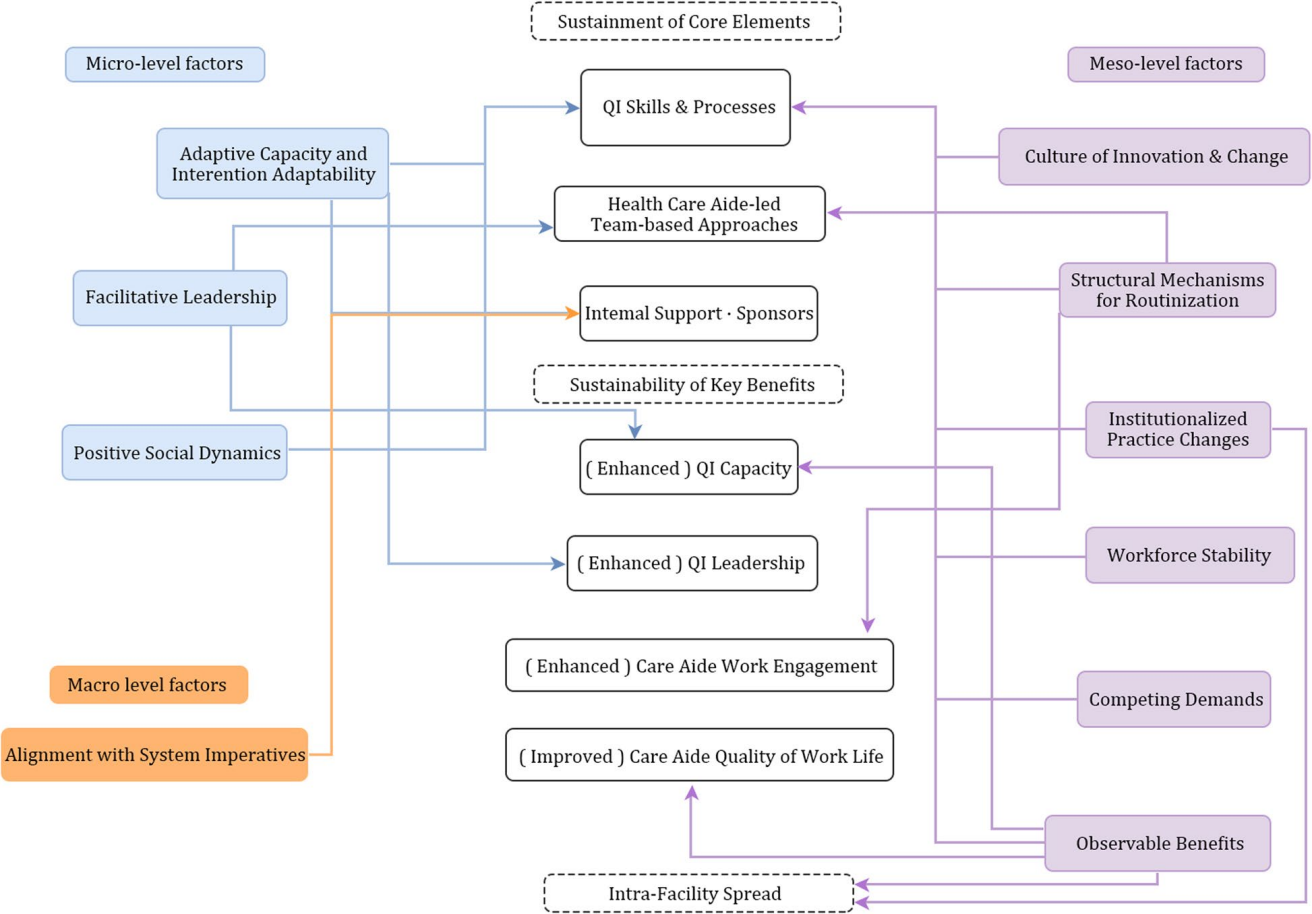
Relationships among Post-Implementation Factors & Sustainability Phenomena

For the post-implementation factors at micro-, meso-, and macro-levels, many relate to sustainment of SCOPE core elements. Some are differentially associated with sustainability and inter-unit spread. Figure 1 summarizes the relationships among post-implementation factors and sustainability phenomena.

#### Micro-level factors

##### Critical QI capacity of care aides and Sponsors is associated with sustainment and sustainability

QI skills and processes conveyed to care aide teams are core intervention elements (Table 1). Interview participants saw opportunities to apply skills and processes post-implementation as important to sustainment and sustainability of benefits. Opportunities arose through QI leadership by care aides (a sustained key benefit) and continued internal support by Sponsors (a sustained core process). The CH3 Senior Sponsor illustrated the importance of combined opportunities and support. One care aide persistently engaged colleagues in applying the PDSA cycle. This engagement was reinforced by continued Sponsor support through open communication and active facilitation.

##### Adaptive capacity of care aides and adaptability of the intervention facilitate sustainment

Sponsors from three CHs (CH1,3,5) observed that SCOPE team members continued to use core QI skills and processes (e.g., PDSA cycles, pre/post measurement, team-based approaches to QI opportunities) post-implementation. These were, however, adapted from the original intervention. Sponsors described adaptations as critical to sustainment. For example, a PDSA cycle approach developed post-implementation in one CH relied on less formal means of identifying problems, trialing small-scale solutions, and assessing results (Table 3 Quote 5).

Sponsors asserted that adapted approaches fit better with care aides’ typical reliance on verbal exchanges with co-workers about residents’ needs and health. This underscores concurrent importance of care aides’ adaptive capacity and adaptability of the SCOPE intervention itself.

##### Facilitative formal leadership of Sponsors is important to sustainment and sustainability

Most Sponsors perceived their continued support for care aides as key to SCOPE continuation. To continue supporting care aide engagement in and leadership of QI initiatives, Sponsors adapted their leadership behaviors. Post-implementation, Sponsors described new initiatives to formally recognize care aides for their QI work (CH1,5), facilitate team activities and encourage open communication (CH3), and highlight CH-wide the importance of care aide efforts (CH4). In CH2, the only home in which sustainment and sustainability were not observed, Sponsors’ post-implementation support for care aides diminished with competing demands. This presented sustainment challenges for care aides, who required Sponsor endorsement to continue work outside their usual role and pursue QI.

Sponsors keenly felt significant changes to ways they led: adopting new behaviors (facilitating – not leading – QI initiatives) and embracing uncertainties (with some discomfort) in trial-and-error learning (Table 3 Quote 6). Sponsors saw their role as encouraging care aides to take initiative. All these behaviors were acknowledged as departing from top-down leadership norms typical of long-term care.

Effective support also required Sponsors to adjust their behaviors to care aides’ fluctuating needs. For example, the CH4 Team Sponsor took a less directive approach to encourage care aide leadership but was intermittently more directive when QI team momentum faltered (Table 3 Quote 7).

While Sponsors acknowledged that care aides’ QI skills were essential to sustain core SCOPE elements, a few remarked on organizational skills and care aides’ ability to look beyond task-driven work. CH2 Team and Senior Sponsors attributed additional reasons for failure to sustain SCOPE to care aides’ apparent lack of skills in QI team organizing and inability to think beyond tasks to bigger picture QI initiatives. This signals real tension from competing demands on care aides and infeasibility of addressing higher order demands if care aides lack overt management support to step outside usual roles.

##### Social dynamics among Sponsors and care aides are important to sustainment

Sponsors shared examples of positive social dynamics peri-implementation that were important to continuing core elements and key benefits post-implementation. Dynamics facilitated sustainment through interactions between Sponsors and care aides and interactions among care aides. The CH3 Senior Sponsor specifically referenced the importance of trust to sustaining proactive QI communication and activity (Table 3 Quote 8).

Post-SCOPE, the CH4 Team Sponsor and the remaining 3 SCOPE care aides worked on different units but exchanged ideas during breaks. The Team Sponsor cited this as the source of sustained momentum and post-implementation sustainment of SCOPE-related activities. Indirect interactions between CH visitors and care aides motivated project continuation (Table 3 Quote 9).

#### Meso-level factors

##### Culture of innovation and change is associated with sustainment

Participants associated unit cultures open to innovation and change with post-implementation sustainment of QI processes. Some described the culture of innovation and change as prevailing before SCOPE and reflecting attitudes toward knowledge use for positive practice change (Table 3 Quote 10).

Less experience with and openness to change negatively affected ability of QI teams to sustain core processes post-implementation. CH2 Sponsors described teams struggling to engage others on their unit. Their QI initiative was seen as unnecessary extra work. Challenges continued post-implementation. Culture and staff attitudes to change were described as “*making or breaking*” post-implementation sustainment. Infrequently but notably, Sponsors described staff outside a QI team resisting activities initiated by team member peers, seemingly because activities were counter-cultural and challenged established routines.

##### Structural mechanisms are associated with sustainment and sustainability

Some Sponsors associated structural mechanisms within the unit and their CH with sustainment and sustainability. Sponsors intentionally introduced these mechanisms to routinize processes and behaviors, including team-based approaches to care and QI approaches to problems. In team, unit, and CH forums, staff could ask QI teams about initiatives, which sustained interest and momentum. One Senior Sponsor described additional benefits to teams of staff recognition and sustained engagement, with QI as a standing agenda item for staff meetings (Table 3 Quote 11).

##### Institutionalized practice changes contribute to sustainment and spread

Formally embedding evidence-based practice changes in care aides’ daily work routines was one means to sustain changes. Participants offered examples of “fixed” changes to mealtime environment and resident care processes that were still in place two years post-implementation. The CH1 Senior Sponsor observed impact of embedding practice changes on both sustainment and spread of a mobility initiative to other units (Table 3 Quote 12).

##### Workforce stability influences sustainment

Both *team member* and *formal leadership* stability were identified as important. Sponsors from CH1 and CH3–6 noted at least one original SCOPE care aide team member who remained with each original participating unit. Sponsors described them as keepers of institutional knowledge on core SCOPE elements who were essential to sustainment. For example, 2 CH1 care aides from the original team still relied on team-based approaches, organizing regular huddles to develop and track success of QI solutions to identified problems on their unit.

Care aide persistence was helpful but insufficient to guarantee sustainment of QI activities. Stability of Sponsors was seen as crucial to sustaining overall momentum in using core processes. The CH4 Team Sponsor described turnover of their original Senior Sponsor as a serious challenge to continuing activities post-implementation. They were unable to sustain financial support for their QI ideas once the Senior Sponsor left the CH.

##### Competing internal demands detract from sustainment

Some Sponsors described competing demands for care aides’ time and energy, hindering ability to sustain post-implementation QI processes. Care aides often worked multiple jobs, limiting full engagement in “extra” QI work and challenging their ability to gain peer support.

Sponsors also experienced numerous demands. The CH2 Senior Sponsor cited these when reflecting on why their SCOPE QI project was not sustained (Table 3 Quotes 13 & 14).

##### Observable benefits are associated with sustainment, sustainability, and spread

Most Sponsors described noticeable benefits at unit or CH levels – including reduced staff workload and improved resident quality of life – from practice changes through SCOPE. In some instances, observable benefits stimulated buy-in of care aides outside the QI team, contributing to overall post-implementation QI capacity in the unit or CH and continued practice changes (Table 3 Quote 15).

Seeing benefits was important to post-implementation inter-unit spread of core processes and change ideas originating from QI teams. CH4 Sponsors and original QI team members proactively presented and highlighted observable improvements in resident care and in work outcomes like reduced workload. This opened conversations for spreading initiatives to other staff and units (Table 3 Quote 16).

#### Macro-level factors

##### Alignment with system imperatives affects sustainment and sustainability

CH4 and CH6 Sponsors commented that resource constraints in long-term care created competing demands in CHs that reduced their ability to support staff QI initiatives post-implementation (Table 3 Quote 17).

Participants saw QI as one of many initiatives competing for resources. One means to reconcile competing internal and external imperatives was to align CH operational and provincial priorities. Where this was possible, related processes and benefits were sustained (Table 3 Quote 18).

## Discussion and implications

This study focused on understanding unit and facility managers’ perspectives of relationships between post-implementation factors and the phenomena of sustainment, sustainability, and spread of complex interventions. Sustainment of core elements and sustainability of key benefits was observed across most CHs in our study. Spread of the SCOPE intervention was noted in half of the sites observing sustainability and sustainment. Relationships emerged through our analysis of participant interview data.

### Core elements, key benefits, and precedence of influential factors

Factors influencing sustainability phenomena are not well understood [2, 10], for reasons including persistent lack of conceptual consistency, under-specification of complex interventions’ core elements, reductionism leading to limited insight on interrelations among broad influence categories (context, intervention characteristics, processes relating to sustainability, capacity to sustain), and failure to consider factors acting dynamically on sustainability phenomena at different levels within and outside the post-implementation context [2, 8, 18, 30].

Distinctions between core elements, processes, and associated behaviors that are sustained (sustainment) and ensuing benefits (sustainability) are helpful to conceptualize and study sustainability phenomena. Our deep history in developing [22], piloting [24], and implementing [20] SCOPE helped us make distinctions and consider influences of factors active at different levels on sustainment of core elements and sustainability of key benefits. Distinguishing sustainment and sustainability had clear value to our study and may be helpful in other settings and studies of sustainability phenomena.

While we differentiate sustainment and sustainability in analysis levels, the identified post-implementation factors align well with the four broad categories of influence for peri-implementation factors [2]: organizational context (culture, leadership), internal and external capacity, knowledge-conveying and supportive processes, and intervention adaptability. In the broadest sense (discussed further below), our findings contribute understanding of the particular importance of factors. Several micro-level factors and the macro-level factor of system alignment contribute to sustainment of core process elements. Meso-level factors contribute to sustainability of key benefits/outcomes and sustainment of some core processes. Critically, the meso-level factors of observability and practice change institutionalization related exclusively to inter-unit spread.

Our findings suggest multiple influential factors that merit further research. As observed across studies in Stirman and colleagues’ review (2012) [2], and as described by our study participants, changes to *outer context* (e.g., policies, legislation) can alter internal priorities and shift limited resources away from QI initiatives. *Capacity to sustain*, including funding, resources, and workforce stability, is similarly susceptible to external pressures [2]. Even smaller changes to local or regional contexts that influenced management turnover or care aide workforce instability profoundly influenced sustainment of core processes and sustainability of QI capacity and leadership.

### Distinguishing influences of implementation and post-implementation factors

Birken and colleagues (2020) [31] note that research rarely distinguishes between determinants of sustainment and implementation. Our findings suggest that these differ. Specifically, we found that ability to sustain core processes is susceptible to several micro-level factors and vulnerable to vagaries of extra-organizational change. Sponsors perceived competing system priorities as barriers to sustaining and spreading SCOPE-related activities. This was *not* identified as a factor influencing SCOPE’s implementation in our earlier work [19]. Unlike post-implementation, peri-implementation supports (e.g., additional resources, expertise, and focused attention) likely protect the implementation micro-context temporarily from external pressures that discourage internal change and innovation.

### The importance of adaptation

Adaptation is widely acknowledged as an important, but understudied, factor influencing sustainment and sustainability [17, 32]. Here, two aspects of adaptation emerged as important: adaptability of the intervention itself and staff’s adaptive capacity to change the intervention. Sponsors described care aides adapting features of SCOPE elements post-implementation, making features easier to sustain. One unit adapted application of the PDSA cycle, sharing cause-and-effect relationships orally. This viable, reliable alternative to formalized pen-and-paper repeated measures aligned the intervention with context. Reliance on oral communications is characteristic of long-term care and care settings with non-standardized work, complex work environments, and learning by doing [33]. Our findings corroborate observations by other sustainability scholars that “*interplay between contextual factors and the innovation itself is to be expected given the dynamic nature of the complex systems into which innovations are introduced*” ([2], p. 10).

Such observations also relate to fidelity. Partial sustainment is more common than complete sustainment [2]. Adaptation sustains intervention elements that fit with context and discards others. Elements most closely related to desired benefits are more feasibly executed. Prevalence of partial sustainment suggests that fidelity is rarely absolute and raises questions about whether departures from fidelity that achieve partial sustainment – and improve fit – jeopardize intervention integrity. We must distinguish core intervention elements and less critical peripheral ones. Fidelity sufficiency is essential for pragmatic implementation [2].

### Causal pathways to sustainment, sustainability, and spread

Lack of knowledge about causal pathways to sustainment and sustainability is a major acknowledged gap in sustainment and sustainability research [31]. Our study offers insights into these pathways, including evidence of differentiable influences of some factors on particular phenomena and on interrelationships among factors. Research that extends examination of interrelationships among sustainability phenomena and influence factors strikes us and others as promising [7, 25, 32]. Stirman et al. (2012) [2] points to potential for this research to illuminate compensatory factors if elements like funding are missing. We observed the importance of strong social dynamics to sustainment and sustainability, even with diminished dedicated QI support.

Only two factors affecting sustainment and sustainability influenced intra-CH spread of SCOPE core processes: *institutionalized practice changes* and *observability of benefits* from QI initiatives. Spread occurred in only half of study CHs, suggesting that these factors have more nuanced differences in units where spread occurred (e.g., how practices were embedded, how observable benefits were captured or communicated) that warrant further study.

### Dynamism of sustainability phenomena

With this study and earlier work on factors influencing sustainability phenomena [25], we can compare and contrast factors influencing those phenomena at one year and two years post-implementation. This addresses a critique that sustainability is generally examined at a single time point, overlooking its dynamism [2, 18]. All factors influencing sustainability phenomena that were noted at one year post-implementation [22] were also noted in this study two years post-implementation: turnover of key team members, facilitative team dynamics, observable improvements from SCOPE, strong management support of QI teams, buy-in from other CH staff, and (in)adequate resources. However, several additional factors emerged at two years: adaptive capacity of SCOPE teams, adaptability of SCOPE original elements, structural mechanisms for routinization, and institutionalized practice changes. *Adaptive capacity of team* and *intervention adaptability* sustained SCOPE core elements post-implementation. Lack of influence of these factors at one year suggests that peri-implementation research supports (e.g., coaching) may compensate. With lingering effects of external research support removed by two years, intervention fit with organizational context became more important to continuing SCOPE processes.

*Structural mechanism for routinization* and *institutionalized practice changes* both sustained momentum two years post-implementation. During peri-implementation, several SCOPE elements boosted momentum (e.g., exchanging experiences during Learning Congresses, regular coaching) and may have remained high one year post-intervention [19]. Other factors sustaining momentum at two years may have been deemed unimportant at one year.

### Strengths and limitations

#### Strengths

By defining sustainment and sustainability as distinct concepts, we considered both SCOPE’s continued practice and achieved benefits. Previous research suggests that continued practices do not guarantee intended benefits, requiring us to distinguish these concepts [13] and examine both. We interviewed most Sponsors who participated in SCOPE, keeping our findings relevant to local contexts and reflecting CHs with and without sustainment activities.

#### Limitations

First, all sites were from the same region and regulated by the same health authority. They may have more similarities than differences, limiting generalizability of findings. Second, this study used secondary data that were originally collected to inform the SSaSSy study. Secondary data analysis limited our ability to ask follow-up questions or probe deeper on issues related to this study. Also, data from care aides were unavailable. Care aide experiences and perspectives may differ from those of Sponsors, and Sponsors’ perceptions and reports may be positively skewed. However, Sponsors gave insightful administrative overviews. In next phases of the SSaSSy project, we will seek perspectives of both Sponsors and care aides. Third, this retrospective study was susceptible to recall bias. Fourth, we used telephone interviews to collect data. Lacking face-to-face interactions may have made it challenging to establish trust with the interviewees. However, this challenge was mitigated as the managers knew the interviewer (WB) and the research team during their prior participation of SCOPE.

### Contributions to theory and practice

Our foremost practical contribution is demonstrating that *post*-implementation factors are important influencers of sustainment, sustainability, and spread of complex interventions. This suggests that similar interventions in the long-term care sector should be developed with post-implementation sustainability in mind, with measures to protect against influences such as workforce instability and competing internal and external demands. The need to adapt intervention aspects must be anticipated, to enhance post-implementation traction. Participants offered many ways to anticipate vagaries of post-implementation contexts: institutionalizing practice changes, “hardwiring” structural changes that supported process and behavioral changes, highlighting observable benefits, and – whether intentionally or not – building positive social dynamics to keep vital communication channels open and engender staff trust.

Our findings corroborate relationships suggested in extant sustainability theories, models, and frameworks. Many factors identified as influencing sustainment and sustainability related to social interactions among care workers, aligning with well-established implementation science theories that highlight the crucial role of social dynamics among individuals and organizations in implementing and sustaining change interventions [34–37]. Our findings also align with learning theories that underscore importance of institutionalizing changes to practice routines [35]. We identify several unit- and CH-level factors that facilitated integration of SCOPE elements into institutional routines. We observed system-level imperatives that compete with sustaining core elements and key benefits, aligning with open systems theories of organizations as systems profoundly influenced by micro- and macro-environments [37].

Our empirical evidence supports the dynamic view of sustainability [8, 11, 18]. Our findings support the importance for sustainability of constant adaptations and improvement of intervention and organizational context. Facilitative factors for sustainability at two years post-implementation emerged in response to decreasing organization resources for intervention delivery. This corroborates the dynamic sustainability framework, which “*embraces change as a central influence on sustainability*” ([14], p. 7), including intervention–organization fit. That framework also posits the critical role of an organization’s ability to continuously learn and adapt as “*a core value of the implementation setting*” ([14], p. 6). Facilitative factors for sustainability that emerged only by two years post-implementation indicate that CHs can adapt to external environment changes by spontaneously improving organizational context and intervention.

## Conclusions

Our study responds directly to calls for research that attends to conceptual clarity of sustainability phenomena, considers influences of factors at multiple levels simultaneously, and considers these influences in light of sustainment of core elements and sustainability of key benefits of complex interventions [2].

We identify post-implementation factors active at micro, meso, and macro analysis levels that influence sustainability phenomena. This work complements existing research on how peri-implementation factors influence sustainability.

Most sustainability research focuses on interventions with clinicians in public health and community care [6, 11, 30, 38]. Our study contributes understanding of sustainability phenomena in long-term care, which is inarguably complex, under-resourced relative to most care sectors, and reliant predominantly on unregulated healthcare workers with less formal exposure to evidence-informed complex interventions. The fact that complex QI skills and processes were sustained for two years after implementation spotlights under-acknowledged capacity for learning and improvement in this sector.

## Supplementary Information

**Additional file 1.** Interview Guide.

**Additional file 2:** **Supplementary Table 1**. Sustainment, sustainability, and spread by participating care home. **Supplementary Figure 1**. Relationship of SCOPE, data collection for the current study, and SSaSSy. **Supplementary Figure 2**. SCOPE Implementation. 

## Data Availability

The datasets generated during and analysed during the current study are not publicly available due to limitations of ethical approval involving the patient data and anonymity but are available from WB (whit.berta@utoronto.ca) on reasonable request.

## Abbreviations

CH: Care Home
PDSA: Plan-Do-Study-Act
QI: Quality Improvement
SSaSSy: The Sustainment, Sustainability, and Spread Study
SCOPE: Safer Care for Older People in Residential Environments
