# Supplementary material for: Influences of post-implementation factors on the sustainability, sustainment, and intra-organizational spread of complex interventions
Source: BMC Health Serv Res. 2022 May 17;22:666. doi: 10.1186/s12913-022-08026-x (PMC9116057; doi:10.1186/s12913-022-08026-x)
Supplement: Supplementary file 1 — Additional file 1. Interview Guide. [file 12913_2022_8026_MOESM1_ESM.docx]

**Supplementary File 1: Interview Guide**

***Objective:***

To determine how much/if SCOPE-related QI activity is sustained in Winnipeg Care Home units and/or amongst Care Home staff, that/who participated in SCOPE circa 2017.

***Rationale*:**

This will assist us in “assigning” participating Care Homes (units) to booster groups in ALBERTA AND BC (or, at least, knowing what the context is at the beginning of the booster pilot which we anticipate may influence the pilot’s outcomes).

It will also be the basis for a similar questionnaire that we will use to assign to “treatment conditions” (no-, low-, and high- booster).

***Preamble*:**

First, our sincere thanks for agreeing to participate in this follow-up study that aims to focus on how to sustain SCOPE, over the long term, in Care Homes.

As you know, in 2016 a HCA-led team from your Care Home participated in SCOPE, focusing on [add clinical area here]. The SCOPE study concluded in February 2017, and we are interested in learning whether SCOPE-related quality improvement activity continues in your Care Home, what your level of continued involved is in quality improvement, and whether any of the original members of the SCOPE Team continue to be involved in quality improvement in your Care Home.

***Baseline Questions*:**

| **Question** | **Notes** |
| --- | --- |
| **1. Can you describe what your experience was like, participating in the SCOPE study?**  *That is*:  What worked well? Why do you think it worked?  What were the challenges in your Care Home when it came to implementing SCOPE? |  |
| **2. Is there still an active SCOPE Team in your Care Home?** i.e., a group of people who pursue quality improvement projects and use tools and techniques learned during the SCOPE study.  *If not:*  What do you think has led to the team no longer working on quality improvement?  When did the SCOPE Team stop its work?  In your opinion, what are the factors – processes, people, workplace realities, structures, etc. – that discouraged the continued use of SCOPE-related techniques and tools on the part of your HCAs? Were any of these more important than others?  **Thank you for your time.**  *If so*:  In your opinion, what are the factors – processes, people, workplace realities, structures, etc – that have supported the continued work of the SCOPE Team?  Do you still work with the Team as their Sponsor?  What kinds of support do you provide? And, does this differ at all from what you did during the SCOPE study?  Are some or all of the original SCOPE team members still involved (query all staff, including the HCAs, nursing staff, recreational staff, …)?  What do you think has enabled these particular people to continue to work on quality improvement in your Care Home?  *If some members have changed, or are no longer active*:  What has led to changes in team composition? Why have some members continued to work on quality improvement while others have not? |  |
| **3. Do HCAs, specifically, continue to lead quality improvement projects in your Care Home?**  *If not*:  Who now takes the leadership role for quality improvement?  *If so*:  Do these projects focus on quality improvement in the same [clinical area] that was worked on during SCOPE? Or, on another area(s)?  And, have the HCAs:   - been involved in collecting data - measured for outcomes - used the PDSA model - used any of the SCOPE planning documents |  |
