# Supplementary material for: Influences of post-implementation factors on the sustainability, sustainment, and intra-organizational spread of complex interventions
Source: BMC Health Serv Res. 2022 May 17;22:666. doi: 10.1186/s12913-022-08026-x (PMC9116057; doi:10.1186/s12913-022-08026-x)
Supplement: Supplementary file 2 — Additional file 2: Supplementary Table 1. Sustainment, sustainability, and spread by participating care home. Supplementary Figure 1. Relationship of SCOPE, data collection for the current study, and SSaSSy. Supplementary Figure 2. SCOPE Implementation. [file 12913_2022_8026_MOESM2_ESM.docx]

**SUPPLEMENTARY MATERIALS**

**Supplementary Table 1**. Sustainment, sustainability, and spread by participating care home

| **Facility ID** | **Sustainment** | **Sustainability** | **Spread** |
| --- | --- | --- | --- |
| CH1 | √ | √ | √ |
| CH2 |  |  |  |
| CH3 | √ | √ |  |
| CH4 | √ | √ | √ |
| CH5 | √ | √ | √ |
| CH6 | √ | √ |  |

CH = Care Home

**Supplementary Figure 1**. Relationship of SCOPE, data collection for the current study, and SSaSSy


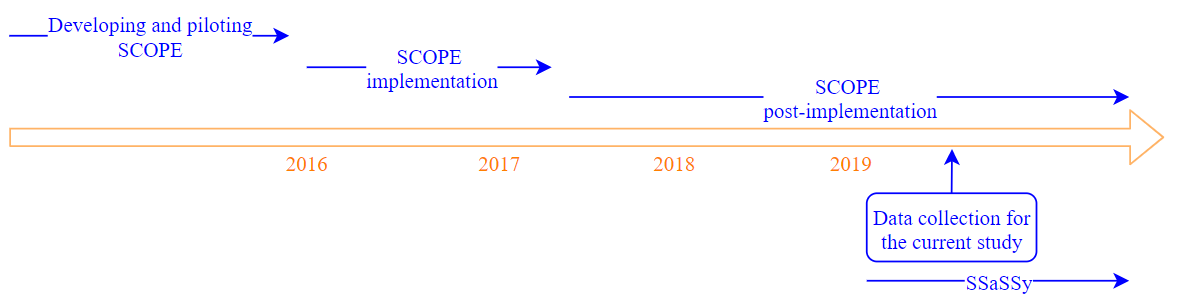


SCOPE = Safer Care for Older People in Residential Environments, SSaSSy = the Sustainment, Sustainability, and Spread Study.

**Supplementary Figure 2**. SCOPE Implementation


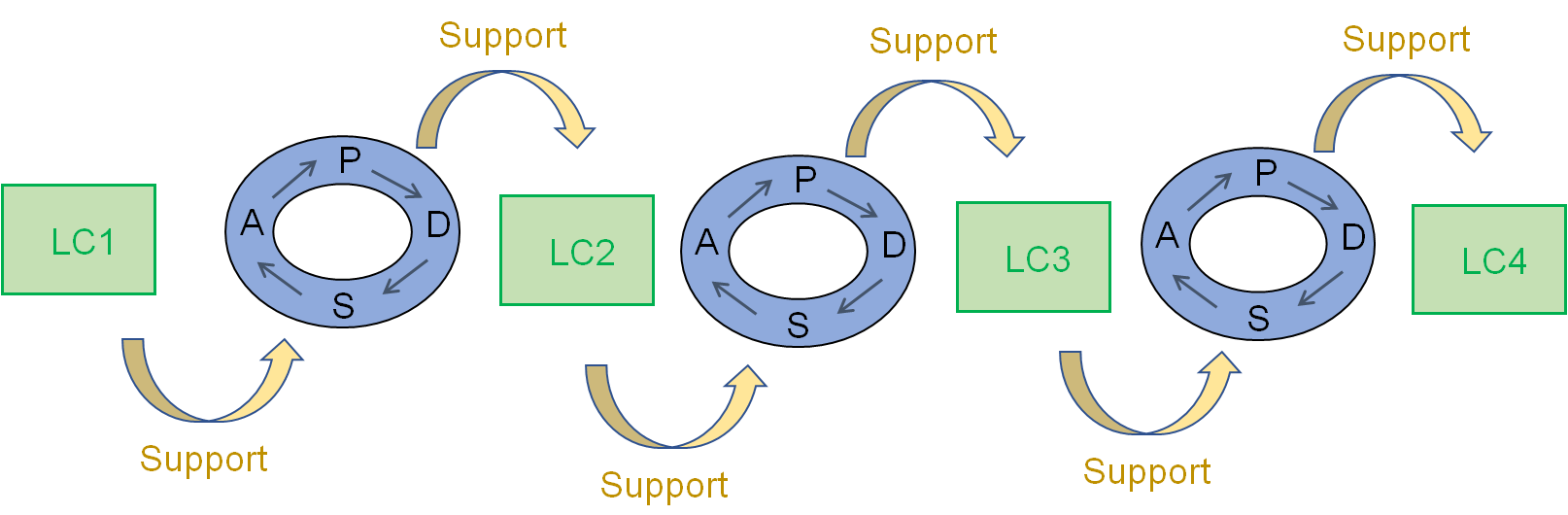


LC = Learning Congress, PDSA = Plan-Do-Study-Act.

Peri-LC support included *internal team support* by Team Sponsors (unit managers) and Senior Sponsors (care home managers) and *external team support* by experienced quality advisors.
